# Loss of melusin is a novel, neuronal NO synthase/FoxO3‐independent master switch of unloading‐induced muscle atrophy

**DOI:** 10.1002/jcsm.12546

**Published:** 2020-03-10

**Authors:** Maurizio Vitadello, Matteo Sorge, Elena Percivalle, Elena Germinario, Daniela Danieli‐Betto, Emilia Turco, Guido Tarone, Mara Brancaccio, Luisa Gorza

**Affiliations:** ^1^ Department of Biomedical Sciences University of Padova Padova Italy; ^2^ CNR‐Institute for Neuroscience, Padova Section Padova Italy; ^3^ Department of Molecular Biotechnology and Health Sciences University of Torino Torino Italy

**Keywords:** Melusin, Muscle unloading, Muscle atrophy, FoxO3, Grp94, gp96, nNOS, Akt, FAK, ERK

## Abstract

**Background:**

Unloading/disuse induces skeletal muscle atrophy in bedridden patients and aged people, who cannot prevent it by means of exercise. Because interventions against known atrophy initiators, such as oxidative stress and neuronal NO synthase (nNOS) redistribution, are only partially effective, we investigated the involvement of melusin, a muscle‐specific integrin‐associated protein and a recognized regulator of protein kinases and mechanotransduction in cardiomyocytes.

**Methods:**

Muscle atrophy was induced in the rat soleus by tail suspension and in the human vastus lateralis by bed rest. Melusin expression was investigated at the protein and transcript level and after treatment of tail‐suspended rats with atrophy initiator inhibitors. Myofiber size, sarcolemmal nNOS activity, FoxO3 myonuclear localization, and myofiber carbonylation of the unloaded rat soleus were studied after *in vivo* melusin replacement by cDNA electroporation, and muscle force, myofiber size, and atrogene expression after adeno‐associated virus infection. *In vivo* interference of exogenous melusin with dominant‐negative kinases and other atrophy attenuators (Grp94 cDNA; 7‐nitroindazole) on size of unloaded rat myofibers was also explored.

**Results:**

Unloading/disuse reduced muscle melusin protein levels to about 50%, already after 6 h in the tail‐suspended rat (*P* < 0.001), and to about 35% after 8 day bed rest in humans (*P* < 0.05). In the unloaded rat, melusin loss occurred despite of the maintenance of β1D integrin levels and was not abolished by treatments inhibiting mitochondrial oxidative stress, or nNOS activity and redistribution. Expression of exogenous melusin by cDNA transfection attenuated atrophy of 7 day unloaded rat myofibers (−31%), compared with controls (−48%, *P* = 0.001), without hampering the decrease in sarcolemmal nNOS activity and the increase in myonuclear FoxO3 and carbonylated myofibers. Infection with melusin‐expressing adeno‐associated virus ameliorated contractile properties of 7 day unloaded muscles (*P* ≤ 0.05) and relieved myofiber atrophy (−33%) by reducing Atrogin‐1 and MurF‐1 transcripts (*P* ≤ 0.002), despite of a two‐fold increase in FoxO3 protein levels (*P* = 0.03). Atrophy attenuation by exogenous melusin did not result from rescue of Akt, ERK, or focal adhesion kinase activity, because it persisted after co‐transfection with dominant‐negative kinase forms (*P* < 0.01). Conversely, melusin cDNA transfection, combined with 7‐nitroindazole treatment or with cDNA transfection of the nNOS‐interacting chaperone Grp94, abolished 7 day unloaded myofiber atrophy.

**Conclusions:**

Disuse/unloading‐induced loss of melusin is an early event in muscle atrophy which occurs independently from mitochondrial oxidative stress, nNOS redistribution, and FoxO3 activation. Only preservation of melusin levels and sarcolemmal nNOS localization fully prevented muscle mass loss, demonstrating that both of them act as independent, but complementary, master switches of muscle disuse atrophy.

## 1 Introduction

Muscle disuse or reduced load, such as occurring to antigravity muscles in bedridden patients, represents a major cause of skeletal muscle atrophy^1^, ^2^. Among the factors responsible for loss of muscle mass and force, a major role has been attributed to the neuronal NO synthase (nNOS) isoform, whose changes in subcellular distribution result in decreased activity of the enzyme at its physiological subsarcolemmal site and increased sustained activity in the myoplasm.^3^, ^4^, ^5^, ^6^ A recent study from this laboratory further demonstrated that nNOS redistribution occurs before the appearance of muscle atrophy and shortly after the exposure to disuse/unloading, that is, after a 6 h unloading bout in the rat soleus muscle and a 8 day bed rest in humans, and is required for the nuclear translocation of the “atrogene” transcription factor FoxO3a.^7^

The unloading‐induced involvement of nNOS/FoxO3 pathway may be prevented by counteracting oxidative stress, both by using generic or mitochondrial‐specific anti‐oxidants^4^, ^7^, ^8^ or by promoting the interaction of nNOS molecules with the endoplasmic reticulum chaperone Grp94/gp96, which improves nNOS maintenance at the sarcolemma.^5^, ^9^ Nevertheless, the adoption of these countermeasures did not fully abolish unloading‐induced myofiber atrophy.^5^, ^8^, ^9^ Consistently, a slight, but significant, degree of myofiber atrophy was observed even when 7–15 day hindlimb unloading was performed after knocking out nNOS or inhibiting its enzyme activity using 7‐nitroindazole (7‐NI)^3^, ^5^.

The partial anti‐atrophic effects obtained by targeting unloading‐induced nNOS redistribution or activity strongly suggest that other pathways, in addition to the one involving nNOS/FoxO3/Grp94, are determinant in the development of disuse/unloading‐induced muscle atrophy. Therefore, the aim of the present study was to investigate the signal transduction pathway bound to the widespread mechanosensitive receptor integrins, focusing on the muscle‐specific transducer melusin.

Melusin is a chaperone protein expressed specifically in cardiac and skeletal muscles. It consists of two N‐terminal cysteine‐rich and histidine‐rich domains: a CS domain (peculiar of cysteine‐rich and histidine‐rich domains and Sgt1 proteins)^10^ and a C‐terminal Ca^2+^‐binding domain, rich in aspartic and glutamic acid residues.^11^ Melusin has been found to interact with the cytoplasmic region of β1 integrin through its CS domain^11^ and to transduce protective signals in the myocardium in response to mechanical stress induced by pressure overload^12^ or myocardial infarction,^13^, ^14^ promoting compensatory left ventricle hypertrophy and cardiomyocyte survival.^15^, ^16^, ^17^ Indeed, mechanical stress activates specific signal transduction pathways in cardiomyocytes, such as the MAPK and the phosphoinositide 3‐kinase (PI3K)/Akt pathways, able to induce a compensatory hypertrophy, crucial in coping with the increased workload. In cardiomyocytes melusin interact with a number of signalling proteins working in these pathways, including the focal adhesion kinase (FAK), the MAPK scaffold protein IQGAP1, the mitogen activated protein kinases c‐Raf, MEK1/2, ERK1/2,^18^ and PI3K.^19^

Melusin is also expressed in skeletal muscles; however, its function in this context has been poorly investigated so far. Early studies indicated that melusin expression is regulated during skeletal muscle development, becoming detectable in embryo limbs at 15 days of gestation and reaching a peak in newborn muscles. In accordance with a possible role in myogenesis, melusin expression is upregulated during muscle regeneration after trauma.^12^ Moreover, melusin has been found overexpressed in muscles from limb‐girdle muscular dystrophy type 2 patients, where it regulates β1D integrin isoform expression.^20^

By investigating rat and human skeletal muscles after short and long periods of unloading/bed rest, here we identify melusin as a new and early disuse target. The rat tail suspension model was further used to monitor melusin expression after exposure to muscle atrophy attenuators such as 7‐NI,^3^, ^5^ or curcumin, which upregulates Grp94/gp96,^9^ or MitoTEMPO, which inhibits mitochondrial‐derived oxidative stress and maintains sarcolemmal nNOS activity,^7^ as well as to evaluate the effects of exogenus melusin replacement, by means of *in vivo* transfection and viral infection. *In vivo* transfections allowed to compare melusin‐transfected myofibers with untransfected ones in the same muscle section concerning myofiber cross‐sectional area (CSA), sarcolemmal nNOS activity by means of histochemistry for NADPH‐diaphorase, FoxO3 nuclear localization, and myofiber carbonylation by means of DNPH adducts. *In vivo* melusin cDNA transfection was further used during unloading in combination with 7‐NI treatments or other constructs, such as dominant‐negative (DN) forms of the melusin‐interacting kinases, or other pro‐trophic players, such as the Grp94/gp96 chaperone. Infections with adeno‐associated virus (AAV) coding for melusin allowed to investigate whether unloaded muscles preserved contractility, in addition to myofiber CSA, composition in fiber types, myosin heavy chains, levels of atrogene transcripts, and FoxO3 and kinase proteins.

The combination of these approaches demonstrates here that melusin is required to maintain mass and force of unloaded muscle. Interestingly, unloading‐induced melusin loss is not rescued by known atrophy attenuators, and melusin replacement appears to counteract atrophy independently from both integrin‐downstream signalling pathways and the preservation of nNOS activity and subcellular distribution.

## 2 Material and methods

### 2.1 Constructs

Exogenous melusin expression was achieved by expressing human melusin cDNA added with a c‐myc tag at the amino‐terminus, either subcloned in a pCEP plasmid or in the AAV 9 (AAV‐MEL).^21^ Empty plasmid (EV) and AAV 9 (AAV‐EV) were used as controls.

Plasmids for DN non‐receptor kinases were provided from Addgene: (i) HA‐Akt‐DN (plasmid 16243) codifies for a mouse Akt variant, which is catalytically inactive due to K179M substitution at the ATP‐binding site and was effective with both rat and human cells^22^, ^23^, ^24^; (ii) pcDNA3‐HA‐ERK2 D319N (plasmid 8979) codifies for a rat ERK2 variant with a mutation preventing the docking (D)‐domain‐dependent ERK binding (D319N) to substrates^25^; and (iii) pEGFP‐RapR‐FAK‐YM‐KD (plasmid 25929) codifies for a murine DN‐FAK form, containing the kinase dead mutation D546R, which was also effective in human cells.^26^

Plasmid pT94, a bicistronic vector containing rabbit Grp94 and GFP cDNAs,^27^ and the corresponding empty vector pT, expressing GFP only (Invitrogen), were also used.

### 2.2 Rat hindlimb suspension

About 136 six week old female Wistar rats were used in the study. Rats entered in each experimental protocol in small groups (*n* = 8–9 in total) and were randomly selected for standard caging (*n* = 2–3) or hindlimb unloading (*n* = 6) using the tail suspension model.^5^, ^7^, ^9^, ^28^ The protocol was performed following the recommendations provided by the European Convention for the protection of Vertebrate Animals used for Experimental and Scientific purposes (Council of Europe number 123, Strasbourg, 1985) and authorized by the Animal Ethics Committee of the University of Padova and the Italian Health Ministry (authorizations103/2007B and 502/2015PR). Each animal was weighed before and after the suspension period. Sacrifice was performed from 6 h to 15 days after entry in the unloading experimental protocol, and soleus muscles were excised, weighed, and frozen in liquid nitrogen, immediately or after muscle mechanics analyses. Samples were stored at −80 °C.

Several different experimental protocols combined standard caging and hindlimb unloading to either pharmacological treatments or *in vivo* gene transfer, that is, direct injection in the soleus muscle of plasmids or AAV particles, or both pharmacological treatments and plasmid injection.

Data concerning number of animals and soleus muscles, together with body and absolute and normalized muscle weight, were reported in Supporting Information, *Table* S1, except for data relative to plasmid‐injected muscles, because of possible flaws on muscle weight due to the transfection procedure.

#### 2.2.1 *In vivo* plasmid transfection

Plasmid transfection was performed as previously described^5^ on about 65 rats, that is, by injecting constructs in the exposed soleus muscle, bilaterally, and applying a train of six 20 ms electrical impulses at 209 V/cm on the closed wounds. The day after, animals were randomly selected for tail suspension for 7 days or let free to ambulate.

The following constructs were used for soleus muscle transfections: (i) about 50 μg of melusin cDNA or EV were co‐injected with 30 μg of pT bilaterally in the soleus muscles of 32 rats, which were then subdivided in four different groups corresponding to (a) freely ambulating animals transfected with EV (*n* = 5); (b) freely ambulating animals transfected with melusin cDNA (*n* = 5); (c) hindlimb‐unloaded animals transfected with EV (*n* = 11); and (d) hindlimb‐unloaded animals transfected with melusin cDNA (*n* = 11); (ii) about 50 μg of each plasmid codifying for DN forms of Akt or ERK2 or FAK were injected in one soleus and in combination with 50 μg melusin cDNA in the contralateral one in 15 rats (five rats for each DN‐kinase plasmid), which were tail‐suspended for 7 days; (iii) about 50 μg of plasmids codifying for melusin combined either with 50 μg of plasmid pT94 or with pT one were injected in each soleus of the same rat. This procedure involved eight rats, which were then tail‐suspended for 7 days.

#### 2.2.2 *In vivo* adeno‐associated virus infections

Rats used for AAV infections were listed in Supporting Information, *Table* S1. After amplification in HEK293 cells, about 1 × 10^10^ MOI of AAV‐MEL or AAV‐EV were injected bilaterally in soleus muscles of 4 week old female rats. A group of rats was sham infected by injecting saline only. From 3 weeks to 15 days after, rats from each group were randomly selected for 7 day tail suspension or standard caging.

#### 2.2.3 Pharmacological treatments

A group of rats listed in Supporting Information, *Table* S1, was treated with 7‐NI (Sigma) or the corresponding vehicle (peanuts oil) by intraperitoneal injection, as previously described.^5^, ^9^ Treatment started the day before tail suspension and was repeated daily. The soleus muscles of two groups of five rats (one group treated with 7‐NI and the other with vehicle) were additionally transfected with 50 μg of melusin, and rats were exposed the subsequent day to hindlimb unloading for 7 days.

Still available frozen ambulatory and unloaded muscles obtained from rats treated with curcumin or MitoTEMPO (*n* of muscles = 18 and 10, respectively, including those obtained from vehicle‐treated rats) were used to determine melusin protein levels. Data concerning these experimental groups were already published .^7^, ^9^

### 2.3 Human biopsies

Biometric data of young male participants to the bed rest campaign organized in July 2007 at the Orthopaedic Hospital of Valdoltra (Koper, SLO), and details on ethical permission have been already published.^7^, ^28^ Bed rest was performed without head‐down tilt for 5 weeks. Biopsies were obtained from the vastus lateralis muscles of each participant at each of the following time points, T0, T8, and T35, corresponding to days after the bed rest start, frozen in liquid nitrogen, and stored at −80 °C. Although the programme included 10 participants, complete three‐biopsy sets were available only from eight subjects. Previous morphometric analyses demonstrated the presence of about 20% muscle atrophy in T35 biopsies compared with pre‐bed rest values, whereas no statistically significant reduction of myofiber size was detected in T8 ones.^7^, ^28^

### 2.4 Western blot

Whole homogenates were prepared by dissolving muscle cryosections as previously described .^7^ Equal amounts of proteins were separated by electrophoresis in reducing and denaturing 10% polyacrylamide gels and transferred to nitrocellulose in the presence of methanol. After saturation, blot strips were incubated with anti‐melusin mouse monoclonal antibody (mAb) clone C3 and anti‐β1D integrin rabbit polyclonal antibody (pAb), as previously described.^18^, ^29^ Anti‐FoxO3 rabbit pAb (Sigma) and anti‐total FAK mouse mAb (clone 2A7, Millipore) were also used. Relative protein amount was calculated after normalization of the densitometric signal to the corresponding amount of serum albumin (SA), for rat muscle, and of actin, for human biopsies, after staining with Red Ponceau or incubation with anti‐GAPDH mouse mAb antibody clone 71.1 (Sigma). Staining for P‐Akt (Ser473) and total Akt, and P‐ERK (Thr202/‐Tyr204) and ERK was performed using appropriate antibodies (Cell Signaling). Relative amount of the phosphorylated protein was calculated after normalization with the densitometric signal of total kinase immunoreactivity. Relative amount of kinases was calculated after normalization with either SA or GAPDH, as described earlier.

### 2.5 Quantitative real time PCR

Total RNA from muscles was isolated using Trizol Reagent (Thermo Fisher Scientific), following the manufacturer's instructions. RNA was reverse transcribed by using the high capacity cDNA reverse transcription kit (Applied Biosystem). Gene expression was evaluated using specific primers designed for Itgb1bp2, Atrogin, MuRF‐1, FoxO1, Foxo3a, and Gapdh as endogenous control (see Supporting Information, *Table* S2). The relative quantity of gene expression was analysed with SDS RQ Study software (Applied Biosystem).

### 2.6 Morphometric analyses

#### 2.6.1 Double immunofluorescence and confocal microscopy

Longitudinal cryosections from ambulatory and unloaded soleus muscles were fixed with 4% buffered paraformaldehyde for 30 min at RT, quenched in bovine serum albumin 1% in phosphate‐buffered saline added with 1% goat serum and incubated overnight at RT with anti‐β1D integrin pAb^29^ and anti‐melusin mAb. After extensive rinses, sections were incubated with a mixture of Alexa 568 and 488 secondary antibodies (Invitrogen) diluted in bovine serum albumin 0.5% in phosphate‐buffered saline and preadsorbed with rat serum to eliminate species cross‐reactivity. Sections were analysed at the confocal microscope Leitz SP5 (Leica Microsystem) using sequential scanning with Helium 485 and Argon 288 lasers. Images were collected at 1024 × 1024 pixels after Kalman 4 normalization.

Transverse cryosections from 7 days tail‐suspended rats, co‐transfected with melusin cDNA or EV together with the GFP‐expressing construct pT or infected with AAV‐MEL or AAV‐EV, were stained with rabbit anti‐dystrophin pAb (Abcam) and/or with mouse anti‐myc mAb clone 9E10 or with anti‐FoxO3 rabbit pAb (Sigma). In this last case, to distinguish myofiber nuclei from interstitial ones, sections were doubly labelled with anti‐α sarcoglycan mouse mAb (Monosan) and nuclei counterstained with DAPI, as previously described.^5^ Slides were observed with a Leitz Axioplan microscope equipped for epifluorescence optics, and data were collected from high magnification micrographs. At least 50 myofiber nuclei were identified for each fiber group per muscle. Percentage of FoxO3‐positive nuclei was calculated separately on the total number of counted myonuclei in transfected or untransfected myofibers.

#### 2.6.2 Immunohistochemistry

Consecutive transverse cryosections from ambulatory and 7 days tail‐suspended rats, transfected or co‐transfected with melusin cDNA or EV, and DN‐kinase constructs, or pTGrp94 or pT, or infected with AAV‐MEL or AAV‐EV, were processed for indirect immunoperoxidase using antibodies specific for c‐myc and HA tags, or EGFP and GFP proteins (Invitrogen) as primary antibodies. Bound antibodies were revealed as previously described .^5^ Additional consecutive sections were labelled for embryonic myosin (My) using BF‐G6 mAb, to discriminate between adult atrophic myofibers from injection‐induced regenerating ones, or for slow My, using BA‐D5 mAb, to distinguish between slow and fast myofibers.^5^

Myofiber size was evaluated by measuring CSA. In some cases, measurements were repeated using minimal Feret's diameter to exclude overestimation secondary to sample misorientation.^7^, ^30^ The CSA value for each myofiber population (transfected–untransfected ones) corresponded to the mean of the values measured from more than 100 fibers of the same unloaded muscle (the experimental unit) and was expressed in μm^2^.

#### 2.6.3 NADPH‐diaphorase histochemistry

Histochemistry for NADPH‐diaphorase to reveal distribution of active nNOS molecules was performed on paraformaldehyde‐fixed cryosections following the previously described protocol.^5^, ^7^ Percentage of myofiber stained sarcolemma was calculated by expressing the length of NADPH‐d positive sarcolemma relative to myofiber cross‐sectional circumference.

#### 2.6.4 DNPH immunohistochemistry

Demonstration of myofiber carbonylation by formation of DNPH adducts was performed on freshly cut cryosections as previously described.^5^ In brief, cryosections were derivatized with DNPH diluted 1:10 (Oxyblot Oxidized Protein Detection Kit, Millipore) and, after saturation, incubated with anti‐DNPH antibodies. Bound antibody was revealed by subsequent incubation with peroxidase‐conjugated secondary antibodies and developed with diaminobenzidine.

Slides were observed with a Leitz Axioplan optic microscope. All morphometric analyses were performed on micrographs, obtained the same day from cryosections of different samples stained on the same glass slide together with sections from a control sample. All measurements were performed independently by two investigators using ImageJ software.

### 2.7 Contractile properties of soleus muscle

The experiments were performed *in vitro* as previously described,^9^, ^31^ using a vertical muscle apparatus (300B, Aurora Scientific Inc.) containing a Ringer solution of the following composition: 120 mM NaCl, 4.7 mM KCl, 2.5 mM CaCl_2_, 3.15 mM MgCl_2_, 1.3 mM NaH2PO_4_, 25 mM NaHCO_3_, 11 mM glucose, 30 μM *d*‐tubocurarine, and pH 7.2–7.4, 30 °C, bubbled with 95% O_2_–5% CO_2_. Muscles were stretched to the optimal length (i.e. the length that allowed maximal tension development in response to a single pulse) and electrically stimulated, by two parallel electrodes, with supramaximal pulses (0.5 ms duration) delivered by a Grass S44 electronic stimulator through a stimulus isolation unit (Grass SIU5). Muscle response was recorded through an isometric force transducer (Harvard) connected to an AT‐MIO 16AD acquisition card (National Instruments), and data were analysed by a specific module of the National Instruments LabVIEW software. Twitch and tetanic tensions were normalized to the muscle wet weight (specific tension, Nxg‐1). Tetanic stimulation was obtained by applying trains of supramaximal stimuli at 80 Hz frequency. Maximum rate of rise of tetanus was also measured (*V*_max_) (N/s).

### 2.8 Electrophoretic separation of myosin heavy chains

Analysis of My heavy chain isoforms was performed by the SDS‐PAGE method previously described, which allows separation of slow from fast My isoforms.^31^ Cryosections of soleus muscles were homogenized and solubilized in SDS‐PAGE sample buffer (62.5 mM Tris, pH 6.8, 2.3% SDS, 5% 2‐mercaptoethanol, 10% glycerol). Muscle protein samples (10 μg each) were electrophoresed on 8% SDS‐PAGE slab gels. Electrophoresis was run for 42 h at 70Volt constant. My heavy chain protein bands were revealed by Colloidal Coomassie blue staining. Isoform percentage composition was determined by densitometry of gels.

### 2.9 Statistical analysis

The experimental unit corresponded to the ambulatory/unloaded muscle. Data were expressed as mean, median, and 5th–95th percentiles with outliers, when using box plots representation, and as mean + SEM, when using histograms.

Statistical analysis between two groups was performed utilizing Student's *t*‐test. Paired Student's *t*‐test analysis was used when comparing average CSA or minimal Feret's diameter values or percentage of FoxO3‐positive myonuclei between transfected and untransfected myofibers of the same muscles. One‐way repeated measure analysis of variance (ANOVA) or within‐subject ANOVA, and Tukey or Bonferroni test for *post hoc* analysis, respectively, were used for multiple comparison. In the case of within‐subject ANOVA, average values of transfected and untransfected myofibers were compared with values obtained from transfected muscles obtained from animals of the same body weight (matched participants). Human data were analysed using within‐subject ANOVA with time as repeated factor. *Post hoc* Bonferroni tests were performed to assess specific differences between times.

0.05 was set as the limit for significance.

Graphs were prepared using the SigmaPlot version 2.0 software (Jandel Europe, Germany).

## 3 Results

### 3.1 Melusin protein and mRNA levels are differently affected by hindlimb unloading

Our experimental model of rat tail suspension induced a statistically significant decrease in soleus muscle mass normalized to body weight (MW/BW), which reached −28% and −40% after 7 and 15 days of unloading, respectively, as previously described^7^, ^28^ and detailed in Supporting Information, *Table* S1. The unloading‐induced decrease in MW/BW and myofiber CSA was apparent only after 2 days of unloading (U2), as reported after extensive analyses which excluded the occurrence of statistically significant myofiber atrophy at 1 day of unloading (U1) (see figure 2 in Lechado i Terradas *et al*. 7).

**Figure 1:**
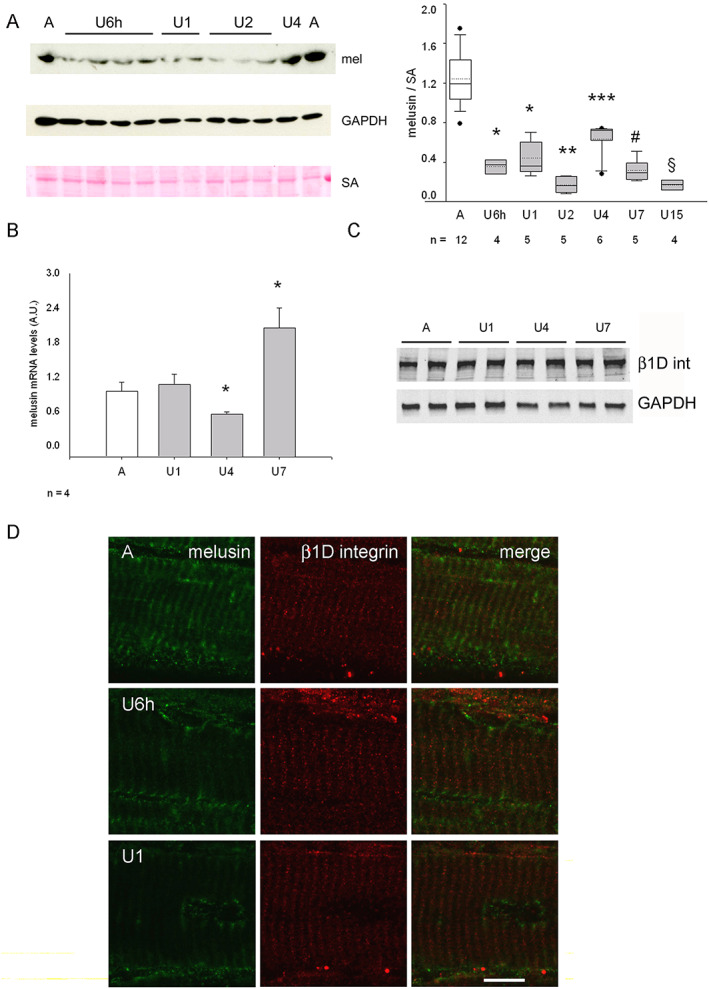
(A) Left panels: representative western blot of whole homogenates from the midbelly region of ambulatory soleus muscle (A) and unloaded (U) ones for 1–4 days, stained with anti‐melusin (mel) antibodies. Red Ponceau staining of serum albumin (SA) and western blot for GAPDH are shown as loading reference. Right panel illustrates box plots with mean (dotted) and median (solid) values of normalized melusin protein levels; *n* indicates the number of animals examined. Single asterisk indicates significant difference to A, U4, U2, and U15 values; double asterisk indicates difference vs. all values except U15; triple asterisk indicates significant difference vs. all; # indicates significant difference to A, U4, and U2; § indicates significant difference vs. all values, except U2 (*P* < 0.001, ANOVA). (B) Histograms show mean and SEM values of normalized melusin mRNA levels from A and U muscles examined at each time point (ANOVA, *P* = 0.001). Asterisks indicate significant difference vs. all. (C) Representative western blot analysis with rabbit polyclonal anti‐β1D integrin antibodies (β1D int, upper panel) and GAPDH (lower panel) on total lysates from A and U muscles. (D) Representative confocal micrographs of double immunofluorescence with anti‐melusin mAb (left column), anti‐β1D integrin pAb (middle column), and merged images (right column) on longitudinal cryosections of ambulatory (A) soleus muscle and after 6 h (U6h) and 1 day unloading (U1). Bar: 10 μm.

Unloading significantly reduced melusin protein levels below 50% ambulatory levels well before muscle atrophy onset, that is, already after a 6 h unloading bout (U6h), as demonstrated by western blotting analyses and normalization with two different loading controls, such as SA and GAPDH (*Figure* 1A and Supporting Information, *Figure* S1). Although the relative protein level of melusin appeared always significantly reduced during unloading compared with ambulatory values, it showed a transient, but statistically significant, stronger decrease at U2 (ANOVA, *P* < 0.0001 and <0.005, after SA and GAPDH normalization, respectively) and again at U15. Quantification of melusin transcripts by quantitative real time PCR showed a significant, transient decrease in mRNA accumulation in U4 muscles compared with ambulatory and U1 muscles and a significant increase compared with both ambulatory and U1 levels at U7 (ANOVA, *P* = 0.001, *Figure* 1B).

At variance with melusin, protein levels of the β1D subunit of muscle integrin did not vary during unloading, as shown by densitometric analyses of parallel western blots (*Figure* 1C, ANOVA, *P* = 0.56; *n* = 5). Such a result was confirmed by confocal microscopy studies (*Figure* 1D). In the ambulatory soleus, labelling for either β1D integrin or melusin was detectable in all the myofibers^32^ (not shown and Supporting Information, *Figure* S2). In longitudinally cut cryosections, both proteins were detected in the correspondence of the Z‐line^12^, ^33^, ^34^, although in the absence of clear‐cut signs of co‐localization. While the distribution of β1D integrin labelling did not change during unloading, melusin staining along the Z‐line decreased strongly already in U6h muscles to disappear almost completely in U1 and U7 muscles (*Figure* 1D and not shown).

**Figure 2:**
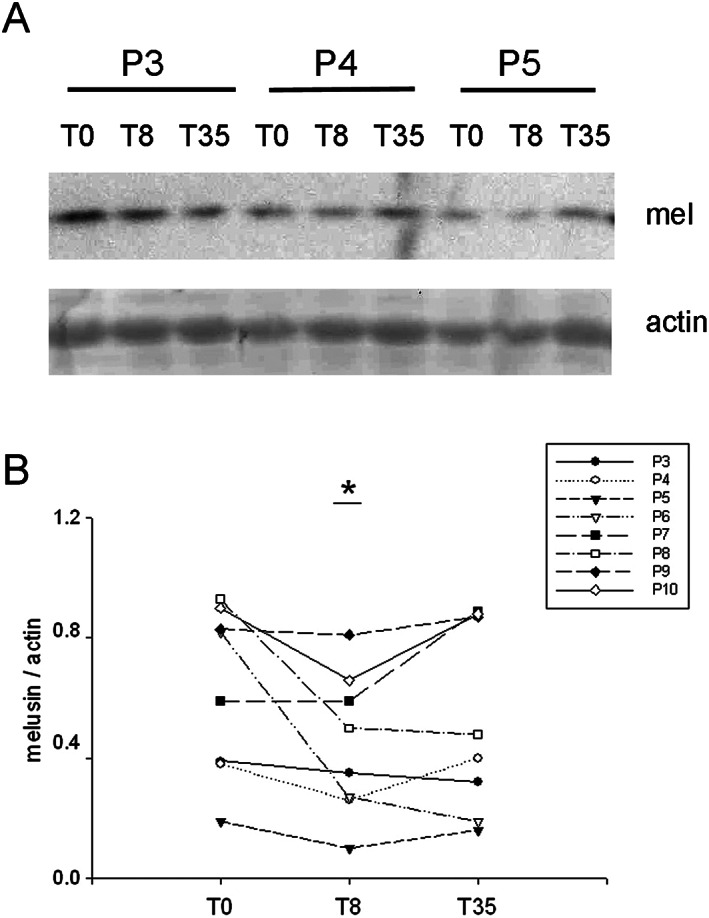
(A) Representative western blot of whole homogenates from vastus lateralis biopsies obtained from three voluntaries (P3, P4, and P5) before (T0) and after 8 days (T8) and 35 days (T35) of bed rest, stained with anti‐melusin (mel) antibodies. Red Ponceau staining of actin is shown as loading reference. (B) Scatter plot illustrates values of normalized melusin protein levels detected in each biopsy. Asterisk indicates significant difference vs. the corresponding T0 value (*P* < 0.05, within‐subject ANOVA).

Similarly, to rat soleus during unloading, melusin protein levels significantly decreased about 35% in human vastus lateralis biopsies after 1 week bed rest (T8) (within‐subject ANOVA, *P* < 0.05, *n* = 8; *Figure* 2), that is, still in the absence of myofiber atrophy,^7^, ^28^ compared with pre‐bed rest levels (T0), whereas they apparently returned to pre‐bed rest levels at T35.

### 3.2 Exogenous melusin expression attenuates myofiber atrophy and loss of force of the unloaded soleus muscle

**Figure 3:**
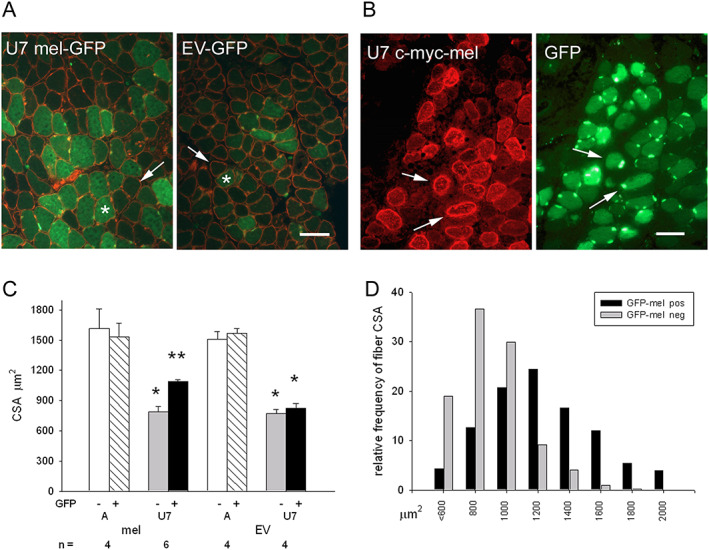
(A) Representative micrographs of 7 day unloaded rat soleus muscle (U7) co‐transfected with either melusin cDNA or empty vector (EV) and a GFP‐expressing construct (green fluorescence) labelled in immunofluorescence with anti‐dystrophin antibodies (red fluorescence). Asterisk and thin arrow indicate representative transfected and untransfected myofibers, respectively. Bar: 100 μm. (B) Representative micrographs of another U7 muscle co‐transfected with melusin cDNA and the GFP‐expressing construct. Myofiber transfection is revealed through labelling for either the c‐myc tag (left panel) or GFP fluorescence (right panel). Thin arrows indicate the presence of both constructs in the same transfected myofibers. Bar: 100 μm. (C) Histograms show mean and SE values of myofiber cross‐sectional area (CSA) in ambulatory (A) and 7 days unloaded (U7) muscles after transfection with either melusin cDNA or EV. GFP+ and GFP− indicate the presence and the absence of transfected cDNAs, respectively; *n* indicates the number of muscles examined; at least 100 fibers were measured for group in each muscle. Single asterisk indicates the presence of significant difference vs. A; double asterisks indicate the presence of significant difference vs. all values (within‐subject ANOVA and Bonferroni's *post hoc* test, ^*^ *P* < 0.005 and ^**^ *P <* 0.05, respectively). (D) Distribution analyses of CSA values of melusin‐transfected myofibers (*n* = 633; black bars) and of untransfected ones (*n* = 595; grey bars) in U7 unloaded soleus muscles (*n* = 6).

In order to determine whether the exogenous replacement of melusin counteracted the atrophy of unloaded myofibers, rat soleus muscles were co‐trasfected with either a c‐myc‐tagged melusin construct or EV, and a GFP‐expressing vector, in order to facilitate the identification of transfected myofibers. Rats were then let free to ambulate or hindlimb unloaded for 7 days (*Figures* 3A and 3B). Results show that unloaded myofibers expressing exogenous melusin displayed a significantly larger CSA, compared with both untrasfected and EV transfected ones (ANOVA, *P* = 0.001, *Figure*s 3C and 3D), myofiber atrophy being attenuated from about −48% to −31%. Apparently, transfection of exogenous melusin in ambulatory soleus did not affect myofiber size (*Figure* 3C).

**Figure 4:**
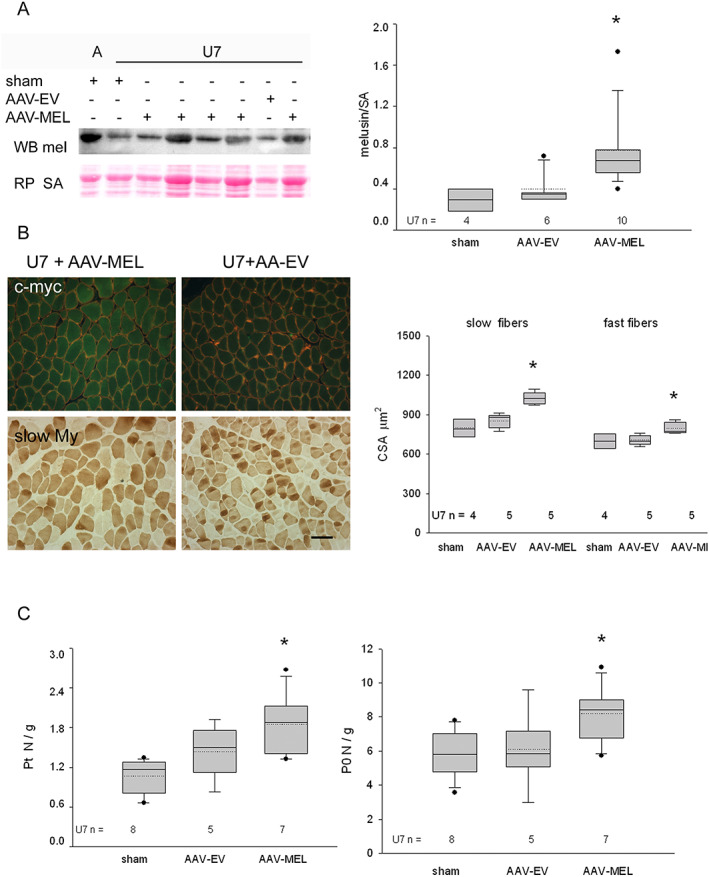
(A) Left panels: western blot stained for total melusin (mel) representatively illustrates the reactivity of ambulatory soleus muscles (A) and 7 days of unloading (U7) ones after sham infection, or infection with AAV expressing melusin (AAV‐MEL) or empty vector (AAV‐EV). Red Ponceau staining of serum albumin (SA) is shown as loading reference. Right panel illustrates box plots with mean (dotted) and median (solid) values of normalized total melusin protein levels in U7 muscles; *n* indicates the number of examined muscles. Single asterisk indicates significant difference vs. sham‐infected and AAV‐EV‐infected muscles (*P* = 0.01, ANOVA). (B) Left panels: representative micrographs of consecutive cryosections U7 muscles infected with AAV‐MEL or AAV‐EV. Upper panels show double labelling with anti‐c‐myc antibody (green fluorescence) and anti‐dystrophin antibodies (red fluorescence); lower panels show immunoperoxidase staining with anti‐slow myosin (My) antibody (dark fibers). Bar: 100 μm. Right panel shows box plots of cross‐sectional area (CSA) of slow and fast myofibers in sham‐infected, AAV‐MEL‐infected, and AAV‐EV‐infected muscles after 7 days of unloading. Solid and dotted lines in boxes indicate median and mean values, respectively; *n* indicates the number of muscles examined; at least 100 fibers were measured for group in each muscle. Single asterisk indicates the presence of significant difference vs. sham and AAV‐EV values of the same fiber population (ANOVA, *P* = 0.001 and 0.01 for slow and fast fibers, respectively). (C) Box plots show mean (dotted) and median (solid) values of normalized twitch and tetanic tension, left and right panels, respectively, in U7 muscles after sham infection, infection with AAV‐EV or with AAV‐MEL; *n* indicates the number of examined muscles. Single asterisk indicates the presence of significant difference vs. sham‐infected muscles (*P* = 0.03 and 0.05, respectively, ANOVA).

To ascertain whether the anti‐atrophic effects of exogenous melusin attenuated the loss in contractile force of unloaded muscles too, soleus muscles were infected with either AAV‐MEL, or AAV‐EV, or saline, before exposure to 7 days of hindlimb unloading. We used an AAV‐MEL MOI dosage which significantly raised mean total melusin levels of U7 muscles compared with sham‐infected or AAV‐EV‐infected U7 ones (ANOVA, *P* = 0.01, *Figure* 4A), but within levels comparable with those observed in sham‐infected ambulatory soleus. In fact, mean and SEM of normalized relative melusin levels of unloaded AAV‐MEL‐infected muscles and ambulatory sham‐infected and EV‐infected ones (*n* = 4 for each group) were 0.77 ± 0.11, 0.80 ± 0.13, and 0.77 ± 0.15, respectively. Infection with AAV‐MEL apparently involved every muscle fiber and significantly increased MW/BW (Supporting Information, *Table* S1) and CSA of U7 muscles compared with sham‐infected and EV‐infected ones (*Figure* 4B; *P* ≤ 0.01, ANOVA), without apparently affecting the relative percentage of fast and slow fiber populations and My heavy chains (Supporting Information, *Figure* S3).

Mechanical studies performed in unloaded muscles showed that infection with AAV‐MEL significantly ameliorated force development, because higher twitch and tetanic tensions were observed after normalization with MW (ANOVA, *P* = 0.03 and 0.05, for twitch tension and tetanic one, respectively, *Figure* 4B). In addition, the infection with AAV‐MEL also significantly ameliorated the maximum rate of rise of tetanus (*V*_max_) of U7 soleus muscle. In fact, *V*_max_ of ambulatory soleus was 3.65 ± 0.29 N/s and significantly decreased to 1.43 ± 0.15 N/s (*P* < 0.001) in U7 and recovered to 2.56 ± 0.20 N/s (*P* < 0.001) in AAV‐MEL treated U7 muscles.

### 3.3 Melusin pro‐trophic effect is independent from kinase pathways

**Figure 5:**
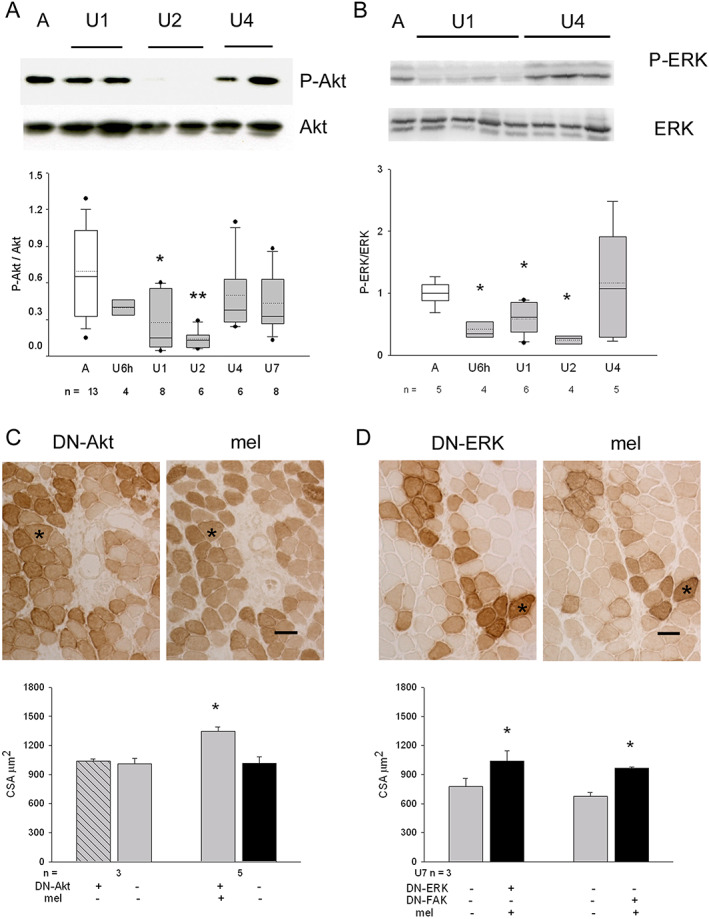
(A) Upper panels show a representative western blot of whole homogenates from ambulatory soleus muscle (A) and unloaded (U) ones for 1–4 days, stained with anti‐P‐Akt and total Akt antibodies. Lower panel illustrates box plots with mean (dotted) and median (solid) values of P‐Akt/Akt ratio; *n* indicates the number of animals examined (*P* < 0.01, ANOVA). Single asterisk indicates significant difference vs. A values; double asterisk indicates difference vs. A, U6h, U4, and U7 values. (B) Upper panels show representative western blots of two parallel gels loaded with whole homogenates from the same A and U muscles and stained with anti‐P‐ERK and total ERK antibodies, respectively. Lower panel illustrates box plots with mean (dotted) and median (solid) values of P‐ERK/ERK ratio; *n* indicates the number of animals examined (*P* = 0.04, ANOVA). Single asterisk indicates significant difference vs. A values. (C) Upper panels show indirect immunoperoxidase of consecutive cryosections from a 7 day unloaded soleus co‐transfected with DN‐AKT and melusin cDNA and stained with anti‐tag antibodies (HA for DN‐AKT or c‐myc for melusin). Asterisk indicates a representative doubly transfected myofiber. Bar: 50 μm. Lower panel: histograms show mean and SEM values of myofiber cross‐sectional area (CSA) measured in positive and negative myofiber populations of U7 muscles transfected with both constructs or with DN‐Akt only; *n* indicates the number of muscles examined. About 100 myofibers for group were considered for muscle. Asterisk indicates significant difference vs. all (*P* < 0.001, within‐subject ANOVA). (D) Upper panels show indirect immunoperoxidase of consecutive cryosections from a 7 day unloaded soleus co‐transfected with DN‐ERK and melusin cDNA, stained with anti‐tag antibodies (HA for DN‐ERKD or c‐myc for melusin). Asterisk indicates a representative doubly transfected myofiber. Bar: 50 μm. Lower panel: histograms show mean and SEM values of myofiber CSA measured in positive and negative slow myofiber populations of U7 muscles doubly transfected with melusin cDNA and either DN‐ERK or DN‐FAK; *n* indicates the number of muscles examined. About 100 myofibers for group were considered for each muscle. Asterisks indicate significant difference vs. untransfected myofibers (*P* < 0.01 paired Student's *t*‐test).

Melusin, as a member of the integrin signalling machinery,^33^ is involved in the activation of several kinases, among which Akt and ERK1/2, in addition to bind others, such as FAK^15^, ^18^. Unloading significantly reduces Akt, ERK1/2, and FAK activation.^35^, ^36^, ^37^ We found that Akt phosphorylation at Ser473 significantly decreased after 24–48 h of unloading compared with ambulatory muscles, whereas it apparently returned to ambulatory levels since U4 (ANOVA, *P* < 0.01; *Figure* 5A). Comparably, ERK1/2 activation appeared significantly inhibited early after unloading, whereas it did not differ from physiological levels at U4 (ANOVA, *P* = 0.04; *Figure* 5B).

We then hypothesized that the unloading‐induced loss of kinase activation might be consequent to the decrease of melusin protein levels. To ascertain whether exogenous melusin replacement attenuated unloading‐induced myofiber atrophy by counteracting kinase inactivation, constructs codifying for HA‐tagged or GFP‐tagged DN‐Akt, DN‐ERK, and DN‐FAK were transfected alone or in combination with human melusin cDNA in the soleus muscle, and rats were hindlimb unloaded for 7 days (*Figures* 5C and 5D). CSA measurements showed that positive myofibers for both exogenous melusin and each DN‐kinase appeared larger than adjacent untrasfected ones or those transfected with DN‐kinase only (within‐subject ANOVA for DN‐Akt and melusin co‐transfections vs. untransfected and DN‐Akt‐transfected unloaded myofibers, *P* < 0.001, *Figure* 5C; paired and unpaired Student's *t*‐test analyses for either DN‐ERK or DN‐FAK and melusin co‐transfected unloaded myofibers vs. untransfected ones, *P* < 0.01, *Figure* 5D). Comparable results were obtained when minimal Feret's diameter was used to evaluate myofiber size (28.85 ± 0.78 and 24.75 ± 1.32 μm, mean and SEM values for DN‐FAK and melusin co‐transfected unloaded myofibers and untransfected ones, respectively, *n* of muscles = 3; paired and unpaired Student's *t*‐test analysis, *P* ≤ 0.03). The possibility that DN‐kinase forms did not blunt exogenous melusin effects on myofiber CSA, because of increased kinase protein levels secondary to melusin expression, was excluded by comparing protein kinase total amounts among sham‐infected U7 muscles, and AAV‐MEL‐infected and AAV‐EV‐infected ones. No difference was detectable (Supporting Information, *Figure* S4).

### 3.4 Melusin pro‐trophic effect does not interfere with nNOS/FoxO3/Grp94 pathway and is synergistically increased by NOS inhibition or Grp94/gp96 overexpression

**Figure 6:**
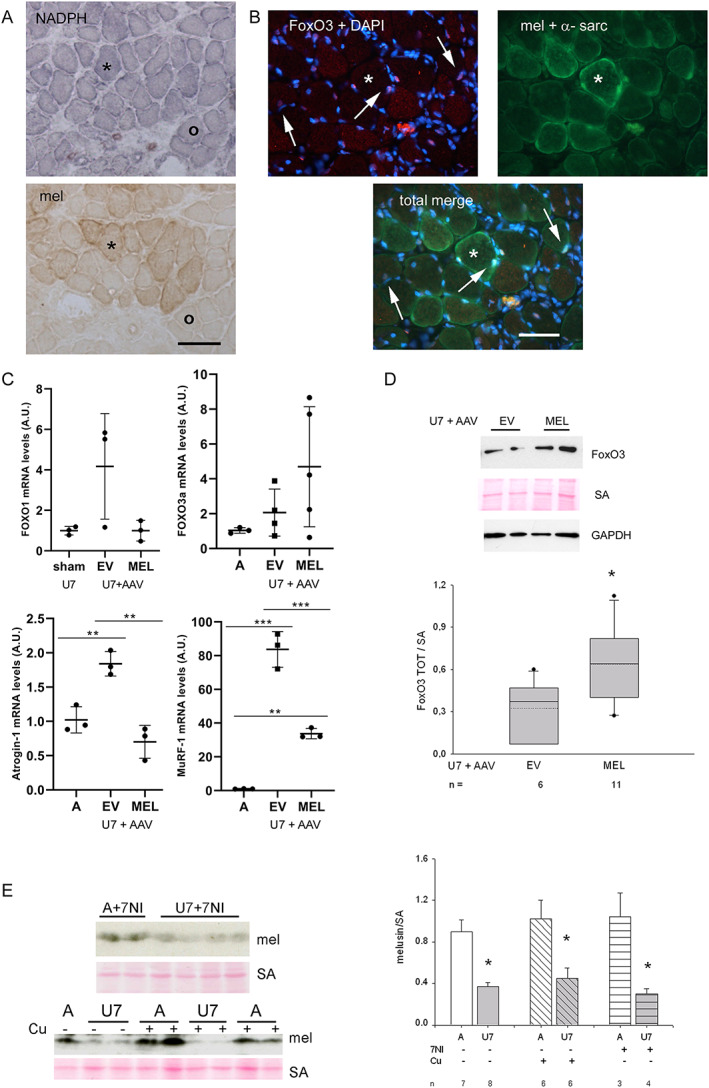
(A) Representative sarcolemmal distribution of active nNOS by means of NADPH‐d histochemistry (upper panel) in melusin‐transfected 7 day unloaded (U7) soleus. Transfected myofibers were identified using anti‐tag c‐myc immunoperoxidase in a consecutive cryosection (lower panel). Asterisk identifies a transfected myofiber, whereas the void circle an untransfected one. Blue formazan staining of sarcolemma is greatly reduced in both transfected and untransfected myofibers. Bar: 100 μm. (B) Immunofluorescence micrographs from a representative U7 melusin‐transfected soleus. The upper left panel shows the staining with anti‐FoxO3 antibody and DAPI (red and blue fluorescence, respectively), whereas the upper right panel shows green fluorescence corresponding to stainings of c‐myc tag and α‐sarcoglycan (the former labels myofibers expressing exogenous melusin and the latter labels sarcolemma). The lower panel shows the merge of all the stainings. FoxO3‐positive nuclei appear pink in the left panel and their myonuclear nature is revealed after merge of all the stainings (arrows). Asterisk indicates a representative transfected myofiber. Bar: 50 μm. (C) Dot plots show normalized values of four atrogene transcript amount detected in ambulant (A) or sham‐infected, AAV‐EV‐infected, and AAV‐MEL‐infected U7 muscles. No significant differences were present for FoxO1 (*P* = 0.07 or ns, ANOVA) and FoxO3 (*P* = 0.14 or ns, ANOVA), while Atrogin‐1 (*P* = 0.0013, ANOVA; *post hoc* Tukey's test, *P* = 0.0012, between EV and MEL) and MuRF‐1 (*P* < 0.0001, ANOVA; *post hoc* Tukey's test, *P* = 0.0002, between EV and MEL) showed significantly reduced levels in MEL‐infected muscles compared with EV ones. Bars in graphs represent standard errors, and asterisks indicate the presence of significant difference (^**^ *P* < 0.01 and ^***^ *P* < 0.001). (D) Upper panels: western blots of U7 muscles infected with AAV‐EV or AAV‐MEL labelled with FoxO3 antibodies. Red Ponceau staining of serum albumin (SA) and western blot for GAPDH are shown as loading reference. Lower panel shows box plots of values of normalized FoxO3 protein levels. Mean and median values are indicated by a dotted and solid line, respectively; *n* indicates the number of muscles examined. Asterisks indicate significant difference (*P* = 0.03, Student's *t*‐test). (E) Left panels: representative western blots of A and U7 muscles after treatment with 7‐nitroindazole (7‐NI; upper panels) or curcumin (Cu; lower panels) stained with anti‐melusin antibodies (mel). Red Ponceau staining of serum albumin (SA) is shown as loading reference. Right panel shows histograms of mean and SEM values of normalized melusin protein levels in the different conditions; *n* indicates the number of muscle examined. Asterisks indicate significant difference vs. ambulatory muscles (*P* = 0.01, ANOVA).

We therefore investigated whether exogenous melusin expression counteracted the effects of other recognized pro‐atrophic drivers, such as the loss of nNOS sarcolemmal activity and localization, and atrogene activation by nuclear FoxO3 translocation (*Figures* 6A and 6B). NADPH‐d histochemistry was performed to investigate myofiber subcellular distribution of active nNOS molecules in melusin‐transfected U7 muscles. Apparently, no difference was detectable between melusin‐transfected unloaded myofibers and untransfected ones (*Figures* 6A). Measurements of NADPH‐d reactivity at sarcolemma showed that melusin‐transfected myofibers displayed even a slightly but significantly reduced percentage of labelled cross‐sectional sarcolemma than untransfected ones (mean and SEM 30.76 ± 3.52% and 33.46 ± 3.36%, for melusin‐transfected and untransfected myofibers, respectively, *n* = 3 muscles examined; *P* = 0.04, paired Student's *t*‐test). It is likely that such a further reduction in sarcolemmal nNOS activity reflects the increase in myofiber cross‐sectional sarcolemma consequent to the pro‐trophic effect of exogenus melusin expression.

The possible interference of melusin transfection with the unloading‐induced activation of the FoxO3 transcription factor was addressed by measuring the percentage of FoxO3‐positive myonuclei relative to total myonuclei of melusin‐transfected myofibers and untransfected or EV transfected ones (*Figure* 6B). Results showed that melusin transfection did not affect unloading‐induced myonuclear translocation of FoxO3, mean and SEM values for the percentage of FoxO3‐positive myonuclei being 61.50 ± 4.72 and 58.20 ± 5.53, for transfected myofibers and untransfected ones, respectively (*n* = 5 muscles examined; more than 100 myonuclei investigated for muscle, *P* = 0.33, paired Student's *t*‐test).

We then analysed whether and how exogenous melusin affected the atrogene expression (*Figures* 6C and 6D). Quantitative PCR investigations on the transcription factors FoxO1 and FoxO3 and the ubiquitin‐ligases Atrogin‐1 and MuRF‐1 showed that each atrogene transcript significantly increased in soleus muscle during unloading, although with variable kinetic (ANOVA, *P* ≤ 0.002, Supporting Information, *Figure* S5) and that FoxO1, Atrogin‐1, and MuRF‐1 transcripts were all significantly upregulated at U7. Expression of exogenous melusin after AAV infection fully abolished Atrogin‐1 transcript accumulation in U7 muscles and significantly reduced MuRF‐1 transcript levels, compared with U7 muscles infected with AAV‐EV (ANOVA, *P* ≤ 0.002, *Figure* 6C). Conversely, it apparently did not hamper FoxO1 transcript accumulation. Strikingly, but not in a statistical significant manner, expression of exogenous melusin in U7 muscles perturbed FoxO3 transcript levels, which failed to return at ambulatory levels (*Figure* 6C and Supporting Information, *Figure* S5).

Western botting analyses were then performed to clarify whether the perturbation of FoxO3 transcript downregulation in unloaded AAV‐MEL muscles corresponded to increased protein levels (*Figure* 6D). Results showed that the relative amount of FoxO3 protein levels was about two‐fold higher in melusin‐expressing U7 muscles (Student's *t*‐test, *P* = 0.03).

To further prove the independence between melusin and the pathway involving nNOS/FoxO3, we investigated whether already known antagonists of this pathway, that is, 7‐NI treatment or curcumin administration, counteracted the unloading‐induced melusin loss. Results showed that melusin protein levels appeared still significantly decreased in U7 muscles either after treatment with 7‐NI or with curcumin (*Figure* 6C, ANOVA, *P* = 0.01), that is, despite of the attenuation of muscle atrophy consequent to each treatment (Supporting Information, *Table* S1, for 7‐NI‐treated rats and figure 2 in Vitadello *et al*.^9^ for curcumin treatment).

**Figure 7:**
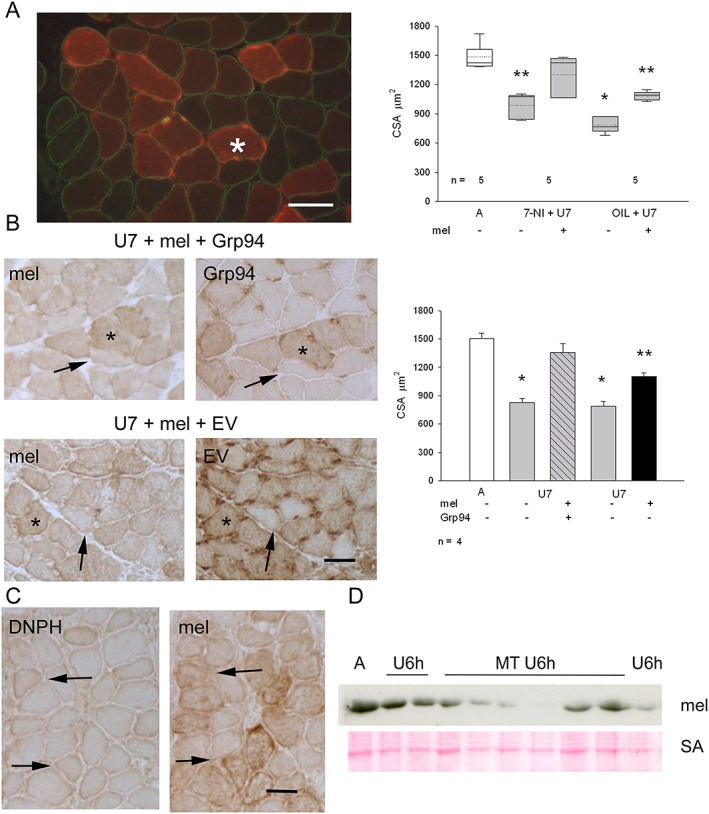
(A) Left panel: immunofluorescence micrograph from a representative U7 melusin‐transfected soleus from a 7‐nitroindazole (7‐NI)‐treated rat stained for c‐myc tag (red fluorescence), to identify melusin‐transfected myofibers, and dystrophin (green fluorescence) Bar: 50 μm. Right panel displays box plots showing mean (dotted line) and median (solid line) values of cross‐sectional area (CSA) of ambulatory (A) untransfected myofibers measured in EV transfected muscles and 7 day unloaded (U7) melusin‐transfected and untransfected myofibers after treatment with 7‐NI or vehicle (OIL); *n* indicates the number of muscles studied. A minimum of 100 fibers was considered per group. Asterisks indicate the presence of statistically significant difference vs. CSA values of A and 7‐NI+U7mel+ (*P* < 0.001, ANOVA and within‐subject ANOVA). *Post hoc* Tukey's test: *P* ≤ 0.004 between A and 7‐NI+U7mel−, OIL+U7mel+, OIL+U7mel−; *P* ≤ 0.05 between 7‐NI+U7mel+ and 7‐NI+U7mel−, OIL+U7mel+ (double asterisk); *P* < 0.001 between 7‐NI+U7mel+ and OIL+U7mel− (single asterisk); *P* = 0.14 between A and 7‐NI+U7mel+; *post hoc* paired Bonferroni's test: *P* = 0.001 between 7‐NI+U7mel+ and 7‐NI+U7mel− and between OIL+U7mel+ and OIL+U7mel− (double asterisks). (B) Left panels: consecutive cryosections from U7 solei co‐transfected with melusin (mel) and Grp94 cDNA or empty vector (EV) stained in indirect immunoperoxidase with anti‐tag antibodies (c‐myc for melusin and GFP for Grp94 or EV). Asterisk indicates a doubly transfected myofiber; thin arrow an untransfected myofiber. Bar: 50 μm. Right panel: histograms show mean and SEM values of A untransfected myofibers measured in EV transfected muscles and U7 myofiber CSA, measured in doubly transfected myofibers and untransfected ones; *n* indicates the number of muscles examined. At least 100 myofibers for group were considered for each muscle. Asterisks indicate significant difference vs. CSA values of A and U7mel+Grp94+ (*P* < 0.001, ANOVA and within‐subject ANOVA). *Post hoc* Tukey's test: *P* < 0.01 between A and U7mel−Grp94−, U7mel+EV+ or U7mel−EV−; *P* ≤ 0.04 between U7mel+Grp94+ and U7mel+EV+ (double asterisk); *P* ≤ 0.002 between U7mel+Grp94+ and U7mel−Grp94−, or U7mel−EV− (single asterisk); *P* = 0.22 between A and U7mel+Grp94+; *post hoc* paired Bonferroni's test: *P* ≤ 0.005 between U7mel+Grp94+ and U7mel−Grp94− and between U7mel+EV+ and U7mel−EV− (single asterisks). (C) Consecutive cryosections from U7 melusin‐transfected soleus stained in indirect immunoperoxidase with anti‐tag antibodies (anti‐DNPH for carbonylated adducts and anti‐c‐myc for melusin). Thin arrow indicates a carbonylated transfected myofiber; large arrow a carbonylated untransfected one. Bar: 50 μm. (D) Western blot of ambulatory soleus muscles (A) and unloaded ones for 6 h (U6h) in the absence or in the presence of treatment with MitoTEMPO (MT) stained with anti‐melusin antibodies (mel). Red Ponceau staining of serum albumin (SA) is shown as loading reference.

Conversely, transfection of exogenous melusin either in combination with 7‐NI treatment (*Figure* 7A) or with co‐electroporation with Grp94/gp96 cDNA (*Figure* 7B) fully antagonized myofiber atrophy of U7 soleus muscles, compared with the attenuation obtained after NOS inhibitor administration or exogenous melusin expression alone (ANOVA, *P* < 0.001; compare also with figures 2C and 7C in Vitadello *et al*. 5), because myofiber size of U7 soleus after each double treatment did not show statistically significant difference from untransfected myofibers of ambulatory muscles (*Figures* 7A and 7B).

Because we demonstrated that unloading‐induced activation of the nNOS/FoxO3 pathway requires a mitochondrial‐derived increase in oxidative stress,^7^ we investigated whether melusin expression levels affected unloading‐induced oxidative stress or were influenced by it. Transfection with exogenous melusin did not apparently counteract myofiber carbonylation of U7 muscles, because the percentage of myofibers reactive for DNPH adducts were comparable between melusin‐transfected and untransfected myofiber populations (*Figure* 7C; mean and SEM values of DNPH‐positive myofiber percentage 20.0 ± 4.8% and 23.2 ± 4.2% for melusin‐transfected myofibers and untransfected ones, respectively, *n* = 3 muscles, about 100 fibers evaluated for each muscle, paired Student's *t*‐test, *P* = 0.11). On the other hand, mitochondrial oxidative stress induced by a 6 h unloading bout did not appear to be responsible for the early decrease in melusin protein amount (*Figure* 7D). In fact, treatment with MitoTEMPO, a specific antagonist of mitochondria‐derived oxidants, did not counteract the unloading‐induced decrease in melusin protein levels (mean and SEM values of melusin densitometric levels normalized to SA signal were 0.28 ≤ 0.08 and 0.40± 0.05 for U6h MitoTEMPO treated (*n* = 6) and untreated muscles (*n* = 4), respectively, Student's *t*‐test, *P* = 0.41).

## 4 Discussion

This study demonstrates that (i) melusin is early involved in the unloading‐induced loss of muscle mass and force and (ii) maintenance of the protein at physiological levels is sufficient to partially counteract disuse muscle atrophy. Indeed, here we show that unloading‐induced muscle atrophy results from the early deregulation of at least two distinct, independent pathways, that is, one leading to melusin loss and the other raising mitochondrial oxidative stress and nNOS subcellular redistribution. Only interventions aimed to prevent both of them fully counteracted the development of muscle disuse atrophy.

Our work on unloaded, yet not atrophic, soleus muscle showed that mitochondrial‐derived oxidative stress represents a required signal driving the myoplasmic redistribution of active nNOS sarcolemmal molecules, which are determinant for FoxO3 nuclear traslocation.^7^ The pioneering work of Suzuki *et al*.^3^ and our previous studies^5^, ^9^ showed that unloading‐induced loss of muscle mass and force was attenuated by counteracting the activation or the myoplasmic redistribution of nNOS molecules. Attenuated atrophy was detected in unloaded soleus from 7‐NI‐treated rats, that is, after NOS inactivation.^3^, ^5^ Comparable attenuation of muscle force and mass loss was also obtained by counteracting the unloading‐induced decrease in expression levels of the endoplasmic reticulum chaperone Grp94, which interacts with nNOS and hampers its redistribution in the myoplasm during unloading,^5^, ^9^ as well as by counteracting oxidative stress, which initiates the redistribution of active nNOS molecules from sarcolemma to myoplasm.^4^, ^7^, ^8^

Here, we identify melusin, the muscle‐specific integrin‐associated protein, as a novel relevant regulator of skeletal muscle mass and force. At variance with nNOS and Grp94, melusin protein levels appear already significantly reduced after a 6 h unloading bout. This event occurs independently from mitochondrial‐derived oxidative stress and appears to be post‐transcriptionally regulated, because downregulation of melusin gene transcription becomes detectable later on. We also show here that the unloading‐induced decrease in melusin protein levels is changed neither after inhibition of mitochondrial derived oxidative stress with MitoTEMPO, nor of nNOS activity by means of 7‐NI, nor after Grp94 upregulation by curcumin administration. Because all these treatments were shown to blunt FoxO3 nuclear translocation/activity,^3^, ^4^, ^5^, ^7^ we may conclude that such an early loss of melusin protein is not the product of FoxO3‐induced atrogene activation.

Furthermore, melusin, but not β1D integrin, is lost from costameres, the macromolecular complexes aligned between the sarcolemma and underlying sarcomeric myofibrils coincident with Z‐discs.^33^, ^34^ Costameres assembly is jeopardized by unloading, because of either decreased FAK activation^37^, ^38^ or reduced expression of the kinase and its inhibitor FRNK, which characterized human muscle already after 8 day bed rest.^39^ Five days of bed rest are sufficient to downregulate transcriptome of integrin signalling pathway in both young and old subjects.^40^ Our finding that melusin, a FAK‐interacting protein, decreased early after unloading also in human muscle is in line with these reports and may represent a major determinant of costamere deregulation.

Indeed, prevention of melusin loss by means of exogenous melusin expression had a beneficial effect in the rat model of muscle disuse/unloading, on both the degree of myofiber atrophy and force development. The pro‐trophic role of melusin has been extensively investigated for cardiac myocytes, where melusin absence accelerates eccentric left ventricle remodelling and transition toward heart failure, whereas melusin overexpression leads to compensatory hypertrophy with increased pump function and protects from dilation.^16^ Our findings in unloaded skeletal muscle confirm melusin pro‐trophic role and show its relevant contribution to contractility. This latter effect, which does not depend only from atrophy attenuation, might result from melusin participation to costamere structure and role in force transmission. Indeed, costameres are hypothesized to collect forces spreading laterally to the long axis of the sarcomere, from each myofibril to the neighbouring one, and to channel them across the sarcolemma to the extracellular matrix, by providing up to 70% of the muscle contraction force.^41^ Thus, the stabilization of the costamere and, as a consequence, of the sarcomere structure and myofilament relationships may explain the attenuated loss of muscle force and contractility. Alternatively, exogenous melusin effects on unloaded muscle contractility may come through the contribution of other proteins. Among possible candidates, Hsp70 was shown to be upregulated in concomitance with melusin overexpression in cardiomyocytes^21^ and effective in counteracting unloading/disuse‐induced loss of muscle mass and force.^42^, ^43^, ^44^, ^45^, ^46^, ^47^ However, such a possibility is apparently excluded here by the lack of significant changes in Hsp70 protein levels in both ambulatory and unloaded AAV‐MEL‐infected muscles compared with AAV‐EV and sham‐infected ones (L. Gorza, unpublished observations).

In addition to contractility, exogenous melusin attenuated the unloading‐induced loss in myofiber size. Melusin pro‐trophic effect occurred acting on ubiquitin‐ligase expression, that is, exogenous melusin expression blunted unloading‐induced Atrogin‐1 transcript accumulation and reduced, without silencing, MurRF‐1 one. Strikingly, such a downregulation occurred in the presence of deregulation of their major inductor FoxO3,^48^ whose protein levels were still significantly higher after 7 days of unloading and maintained a myonuclear localization. Such a result strongly suggests that melusin replacement does not act by silencing FoxO3 signalling pathway. Indeed, exogenous melusin did not hamper nNOS subcellular relocalization or oxidative stress, leading to myofiber carbonylation, two major upstream regulators of FoxO3 activation in the disused muscle.^3^, ^5^, ^7^ Similarly, melusin replacement apparently did not act through the interaction with FAK and the activation of downstream kinases such as Akt and ERK1/2,^12^, ^16^, ^18^ which represent relevant inhibitors of FoxO3 transcriptional activation,^49^, ^50^, ^51^ because co‐transfection of melusin cDNA with defective forms of these kinases did not abolish its pro‐trophic effect. Attenuation of myofiber atrophy by exogenous melusin did not result from the activation of Akt/mTOR/S6K pathway,^35^, ^52^ nor it depended from the parallel activation of the 90 kDa ribosomal S6 kinase, by means of phosphorylation by ERK2 after binding at the D‐domain .^25^, ^52^ Therefore, if FoxO3 is still upregulated and transcriptionally active, the demonstration of Atrogin‐1 silencing and a significant, albeit not complete, MuRF‐1 downregulation suggests the interference of a different pro‐trophic pathway. This possibility is consistent with literature evidence indicating that Atrogin‐1 regulation may occur independently from FoxO3 activation.^48^, ^53^ Therefore, still unknown partners are expected to mediate exogenous melusin signalling leading to Atrogin‐1 silencing in the unloaded soleus muscle.

Consistent with such a hypothesis is the evidence that melusin transfection combined with interventions aimed to block nNOS/FoxO3/Grp94 deregulation, such as treatment with the NOS inhibitor 7‐NI or co‐transfection with Grp94, fully counteracted myofiber atrophy in U7 soleus muscle, implying that both nNOS/Grp94 and melusin are required, because they are independently involved in the maintenance of myofiber size challenged by muscle unloading/disuse.

The possibility that the full pro‐trophic effect resulted from downstream complementation between the two pathways has to be considered too. Increased Grp94 levels may enhance folding and maturation of IGFI and II,^54^ which would act on potentiated IGF receptors by a restored integrin signalling after exogenous melusin expression^55^ and, therefore, abolish myofiber atrophy. However, such a possibility is supported neither by the lack of significant decrease in IGFI and IGFII protein levels in our unloaded muscles^5^ nor by the absence of effects of 7‐NI treatment on the unloaded‐induced decrease of Grp94 protein amount (L. Gorza, unpublished observations). Therefore, our body of evidence indicates melusin as a nNOS/Grp94‐independent player.

Although both nNOS and melusin are costamere proteins, unloading‐induced, mitochondrial‐derived oxidative stress initiates nNOS redistribution in the sarcoplasm,^7^ whereas it does not appear involved in melusin protein decrease. A short unloading bout induces also disorganization in the lipid‐ordered phase of sarcolemma,^56^ detachment of cytoskeletal components, such as the non‐muscle α‐actinin isoform 4,^57^ and loss of function of α_2_Na‐K‐ATPase pump.^58^ It remains to be determined whether any of these unloading‐induced perturbations would act upstream or downstream melusin loss from costameres and catabolism.

In conclusion, this study shows that disuse muscle atrophy results from the deregulation of at least two master switches, melusin and nNOS, only the latter being oxidative stress dependent. In both humans and experimental laboratory animals, the loss of active nNOS molecules from sarcolemma and the decreased melusin amount represent very early events in the development of disuse muscle atrophy. In spite of the desire to uncover a single master regulatory switch, here we demonstrate that unloading‐induced muscle atrophy can be abolished only by addressing simultaneously melusin and nNOS deregulations.

## Author contributions

M.V., M.S., D.D.B., M.B., G.T., and L.G. conceived the experiments and analysed the data; M.V., E.P., M.S., E.T., E.G. carried out experiments. All the authors, except G.T., were involved in writing the paper and had final approval of the submitted version.

## Conflict of interest

All authors declare that they have no conflict of interest.

## Supporting information

Figure S1. A) Representative Western blot of whole homogenates from the midbelly region of ambulatory soleus muscle (A) and unloaded (U) ones for 4 days (left panels) and 7 days (right panels), stained with anti‐melusin (mel) antibodies. Red Ponceau staining of serum albumin (SA) and Western blot for GAPDH are shown as loading reference. B) Box plots with mean (dotted) and median (solid) values of normalized melusin protein levels to GAPDH. n indicates the number of animals examined. Single asterisk indicates significant difference to A values; double asterisk indicates significant difference to A and U6h. (P < 0.005 ANOVA).

Figure S2. Transverse consecutive cryosections from ambulatory rat soleus muscle stained for immunoperoxidase with anti‐melusin mAb (mel) and anti‐slow myosin (slow‐My). Labelling for melusin is detectable in every myofiber with a slight heterogeneity, involving mostly fast myofibers (thin arrow) in addition to slow myofibers (thick arrow). Bar: 50 μm

Figure S3. A) Histograms showing mean and SEM of the percentage of fast fibers in sham‐infected ambulatory (A) and 7‐day unloaded (U) muscles and in AAV‐infected 7‐day U muscles with melusin (U MEL) or empty virus (U EV). N indicates the number of muscles examined. More than 200 fibers were evaluated for muscle. ANOVA P=ns B) Upper panel shows the Coomassie blue staining of a representative gel electrophoresis showing separation of myosin heavy chains (My). Slow My migrates faster than fast My. Lower panels show histograms of mean and SD values of the relative percentage of fast My densitometric values on total ones. ANOVA P = ns

Figure S4. A) Representative Western blots of different whole homogenates from 7‐days unloaded soleus muscles after infection with AAV (U7 + AAV) expressing melusin (MEL) or empty vector (EV) labelled for total Akt and ERK1/2. Parallel staining with anti‐GAPDH antibodies and Red Ponceau staining of serum albumin (SA) is shown as loading reference. B) Left and right panels illustrate histograms of mean and SEM values of normalized total Akt protein levels with SA and GAPDH, respectively. n indicates the number of examined muscles. ANOVA P=ns C) Left and right panels illustrate histograms of mean and SEM values of normalized total ERK1/2 protein levels with SA and GAPDH, respectively. n indicates the number of examined muscles. ANOVA P=ns

Figure S5. Dot plots showing normalized values of four atrogene transcript amount detected in ambulatory (A) soleus muscle and after 1, 4 and 7 days of unloading (U). FoxO1 (P<0.0001, ANOVA; post‐hoc Tukey's test p<0.0001 between A and U7), Atrogin (P<0.0001, ANOVA; post‐hoc Tukey's test p=0.05 between A and U7) and MufF1 (P<0.0001, ANOVA; post‐hoc Tukey's test p<0.0001 between A and U7) transcript were all significantly upregulated at U7. Bars in graphs represent standard errors and asterisks indicate the presence of significant difference (*p < 0.05, **p < 0.01; ***p < 0.001).

Table S1. Body and soleus muscle weights of ambulatory and tail‐suspended rats

Table S2. Primer sets used for qPCR
