# Supplementary figures and images for: Loss of melusin is a novel, neuronal NO synthase/FoxO3‐independent master switch of unloading‐induced muscle atrophy
Source: J Cachexia Sarcopenia Muscle. 2020 Mar 10;11(3):802–19. doi: 10.1002/jcsm.12546 (PMC7296270; doi:10.1002/jcsm.12546)

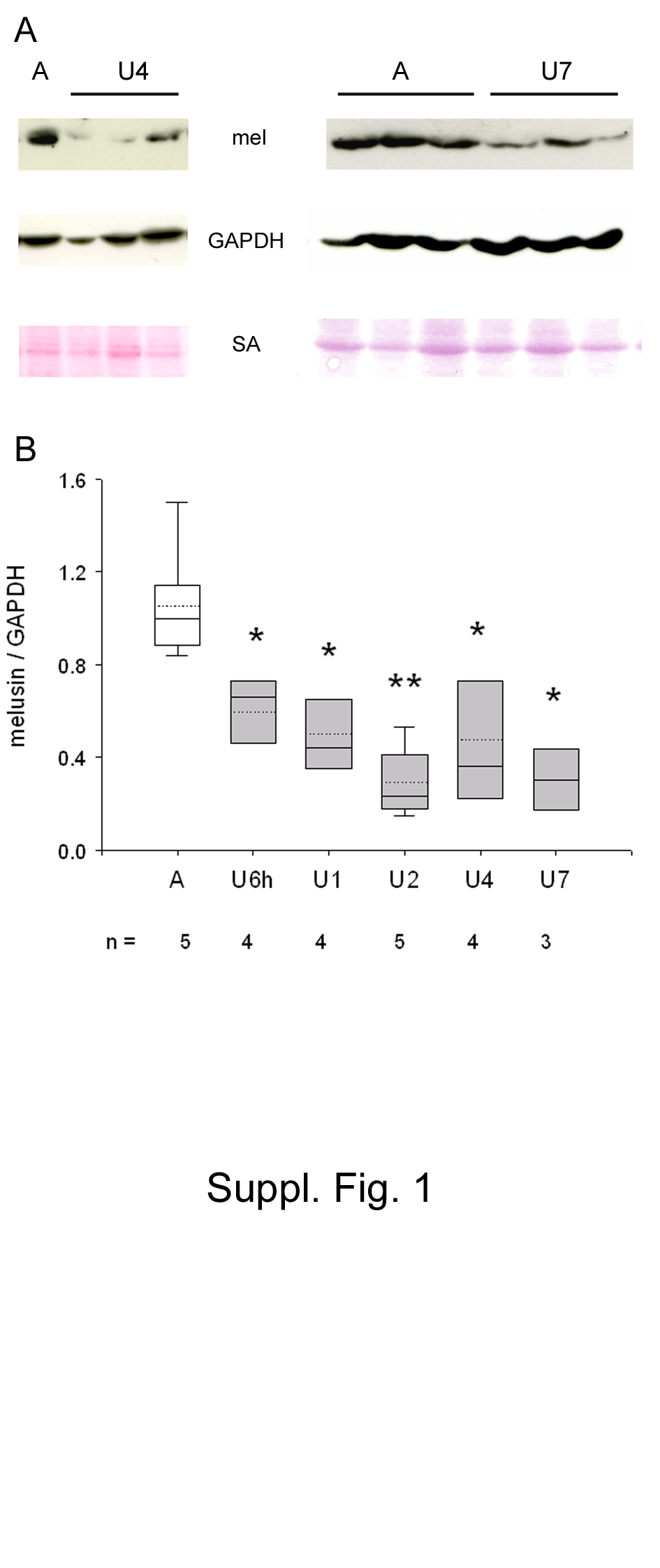

Supplement: Supplementary file 1 — Figure S1. A) Representative Western blot of whole homogenates from the midbelly region of ambulatory soleus muscle (A) and unloaded (U) ones for 4 days (left panels) and 7 days (right panels), stained with anti‐melusin (mel) antibodies. Red Ponceau staining of serum albumin (SA) and Western blot for GAPDH are shown as loading reference. B) Box plots with mean (dotted) and median (solid) values of normalized melusin protein levels to GAPDH. n indicates the number of animals examined. Single asterisk indicates significant difference to A values; double asterisk indicates significant difference to A and U6h. (P < 0.005 ANOVA). [file JCSM-11-802-s001.tif]

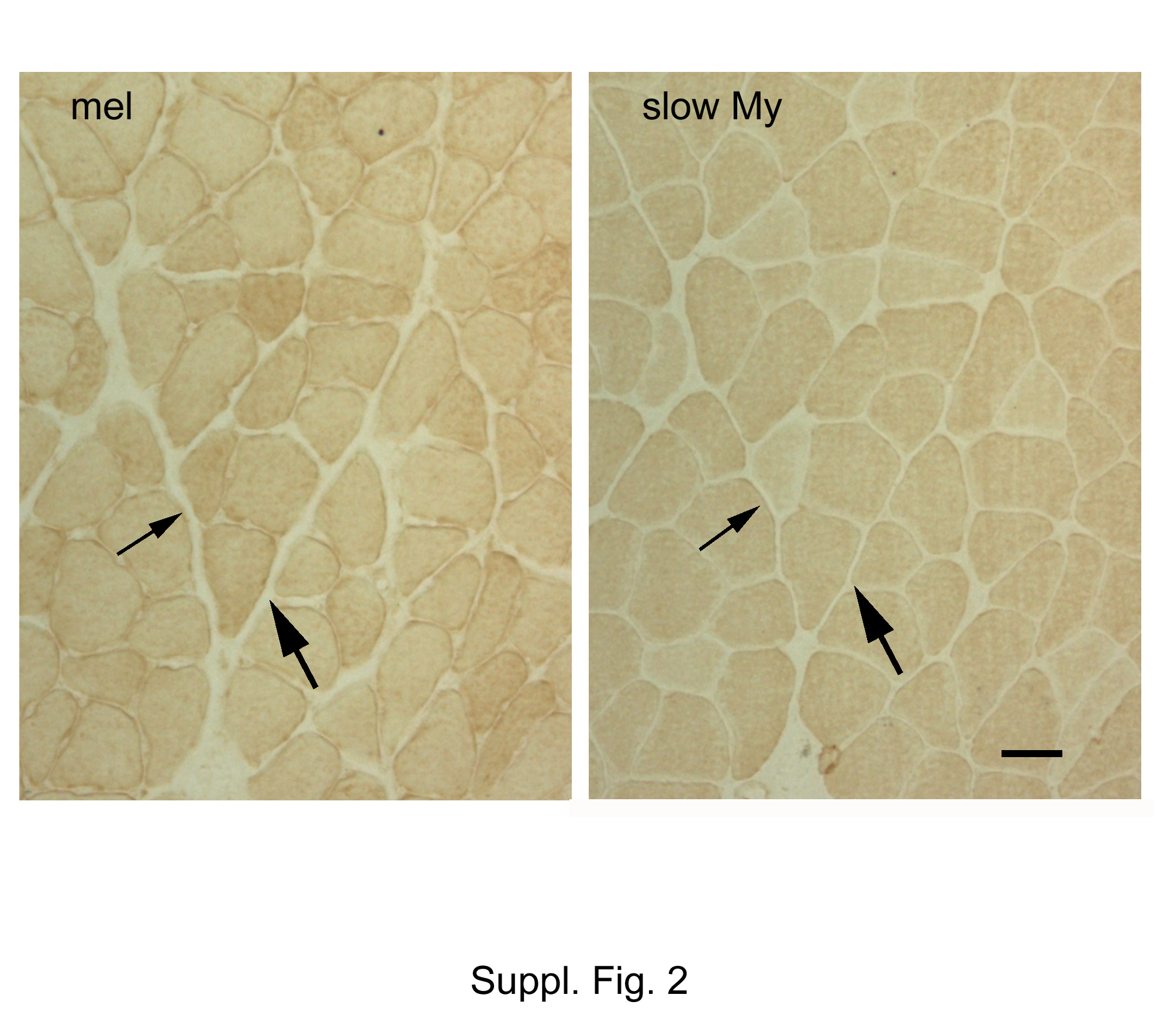

Supplement: Supplementary file 2 — Figure S2. Transverse consecutive cryosections from ambulatory rat soleus muscle stained for immunoperoxidase with anti‐melusin mAb (mel) and anti‐slow myosin (slow‐My). Labelling for melusin is detectable in every myofiber with a slight heterogeneity, involving mostly fast myofibers (thin arrow) in addition to slow myofibers (thick arrow). Bar: 50 μm [file JCSM-11-802-s002.tif]

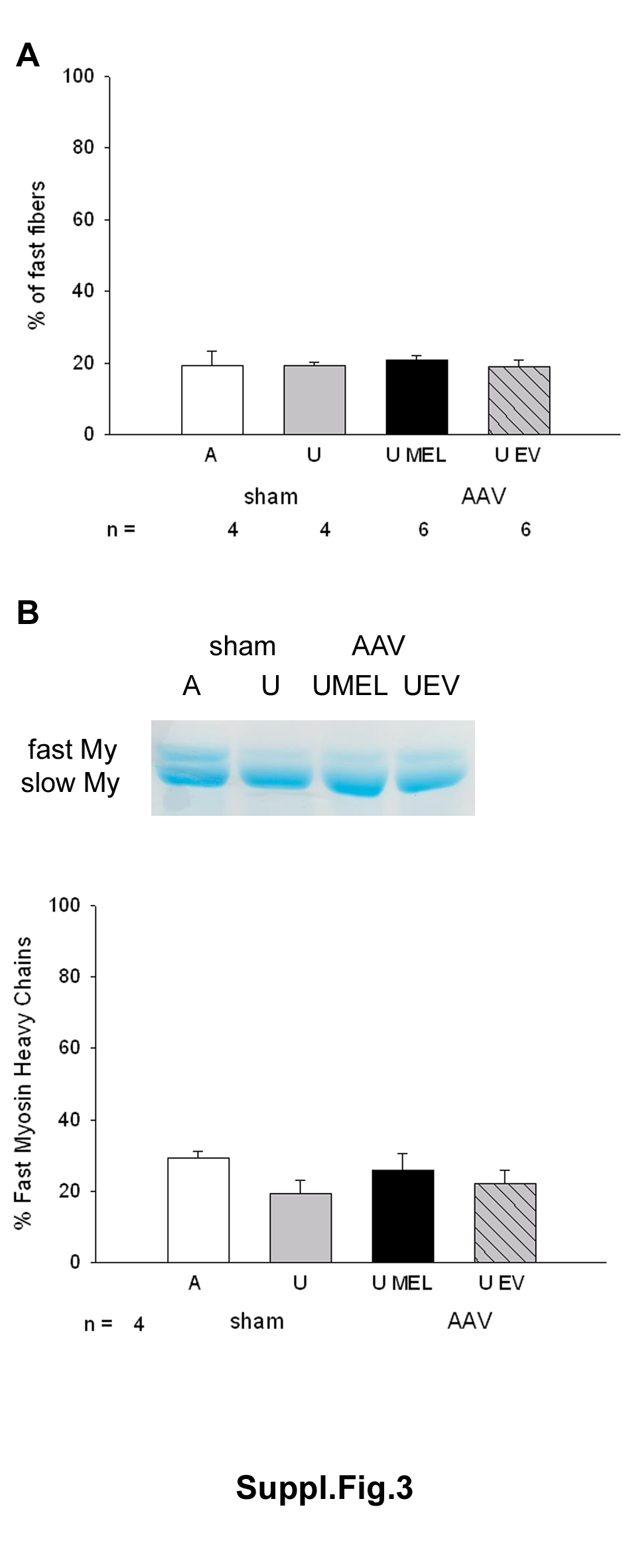

Supplement: Supplementary file 3 — Figure S3. A) Histograms showing mean and SEM of the percentage of fast fibers in sham‐infected ambulatory (A) and 7‐day unloaded (U) muscles and in AAV‐infected 7‐day U muscles with melusin (U MEL) or empty virus (U EV). N indicates the number of muscles examined. More than 200 fibers were evaluated for muscle. ANOVA P=ns B) Upper panel shows the Coomassie blue staining of a representative gel electrophoresis showing separation of myosin heavy chains (My). Slow My migrates faster than fast My. Lower panels show histograms of mean and SD values of the relative percentage of fast My densitometric values on total ones. ANOVA P = ns [file JCSM-11-802-s003.tif]

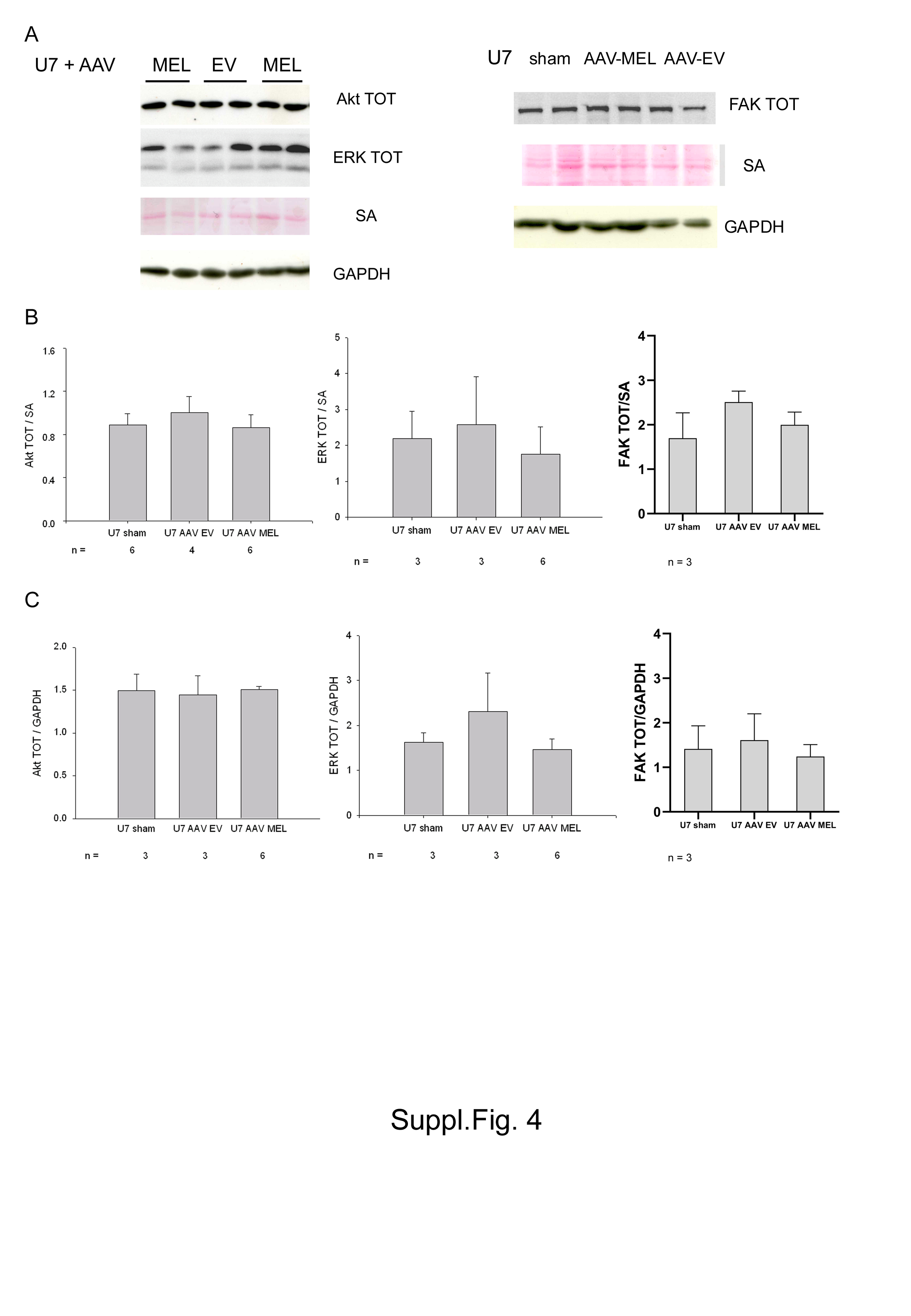

Supplement: Supplementary file 4 — Figure S4. A) Representative Western blots of different whole homogenates from 7‐days unloaded soleus muscles after infection with AAV (U7 + AAV) expressing melusin (MEL) or empty vector (EV) labelled for total Akt and ERK1/2. Parallel staining with anti‐GAPDH antibodies and Red Ponceau staining of serum albumin (SA) is shown as loading reference. B) Left and right panels illustrate histograms of mean and SEM values of normalized total Akt protein levels with SA and GAPDH, respectively. n indicates the number of examined muscles. ANOVA P=ns C) Left and right panels illustrate histograms of mean and SEM values of normalized total ERK1/2 protein levels with SA and GAPDH, respectively. n indicates the number of examined muscles. ANOVA P=ns [file JCSM-11-802-s004.tif]

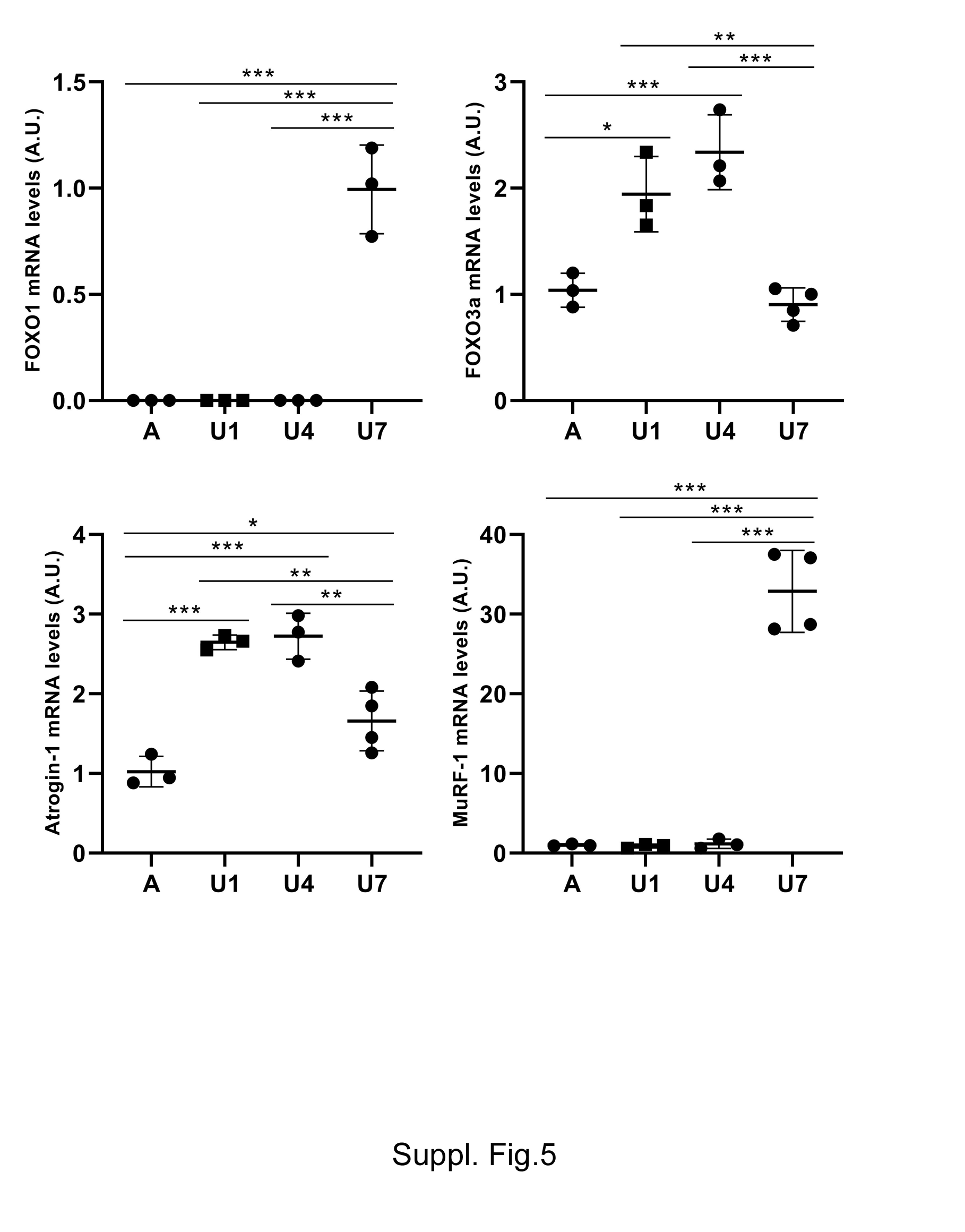

Supplement: Supplementary file 5 — Figure S5. Dot plots showing normalized values of four atrogene transcript amount detected in ambulatory (A) soleus muscle and after 1, 4 and 7 days of unloading (U). FoxO1 (P<0.0001, ANOVA; post‐hoc Tukey's test p<0.0001 between A and U7), Atrogin (P<0.0001, ANOVA; post‐hoc Tukey's test p=0.05 between A and U7) and MufF1 (P<0.0001, ANOVA; post‐hoc Tukey's test p<0.0001 between A and U7) transcript were all significantly upregulated at U7. Bars in graphs represent standard errors and asterisks indicate the presence of significant difference (*p < 0.05, **p < 0.01; ***p < 0.001). [file JCSM-11-802-s005.tif]
