# Supplementary material for: Loss of melusin is a novel, neuronal NO synthase/FoxO3‐independent master switch of unloading‐induced muscle atrophy
Source: J Cachexia Sarcopenia Muscle. 2020 Mar 10;11(3):802–19. doi: 10.1002/jcsm.12546 (PMC7296270; doi:10.1002/jcsm.12546)
Supplement: Supplementary file 6 — Table S1. Body and soleus muscle weights of ambulatory and tail‐suspended rats [file JCSM-11-802-s006.doc]

**Supplemental Table 1 . Body and soleus muscle weights of ambulatory and tail-suspended rats**

| Groups | N of rats  (N of muscles) | BW  (g) | Soleus weight  (mg) | Soleus MW/BW |
| --- | --- | --- | --- | --- |
| **UNTREATED RATS** | |  | | |
| **Ambulatory**^a^ | 13 (13) | 169.0+4.6 | 75.7+4.0 | 0.44+0.01 |
| **Tail suspended** |  | | | |
| 6h | 4 (4) | 148.2+5.7 | 67.5+2.4 | 0.45+0.00 |
| 1 day | 7 (8) | 165.2+6.1 | 73.5+4.0 | 0.45+0.01 |
| 2 days | 3 (6) | 140.3+2.9 | 48.9+2.6* | 0.33+0.02* |
| 4 days | 5 (7) | 164.0+4.2 | 61.4+0.8* | 0.36+0.02* |
| 7 days | 5 (8) | 164.2+6.4 | 52.7+2.0* | 0.32+0.01* |
| 15 days | 3 (4) | 176.0+18.0 | 47.8+5.4* | 0.25+0.03* |
|  |  |  |  |  |
| **AAV-INFECTED RATS** | |  |  |  |
| **Ambulatory**^b^ |  |  |  |  |
| Sham-infected | 2 (4) | 146.0+2.0 | 61.4+1.1 | 0.41+0.00 |
| AAV-MEL | 3 (6) | 150.0+6.5 | 69.5+5.1 | 0.45+0.02 |
| AAV EV | 2 (4) | 150.0+9.5 | 59.3+4.5 | 0.38+0.03 |
| **7-day-tail suspended** |  |  |  |  |
| Sham-infected | 4 (8) | 150.0+4.3 | 41.6+1.3* | 0.27+0.03* |
| AAV-MEL | 6 (12) | 135.5+7.1 | 44.5+1.2* | 0.33+0.01*^§^ |
| AAV EV | 4 (6) | 144.0+7.8 | 44.5+2.4* | 0.30+0.01* |
|  |  |  |  |  |
| **7-NITROINDAZOLE (7-NI) TREATED RATS** | |  |  |  |
| 7NI-Ambulatory | 3 (3) | 154.0+3.0 | 72.8+1.7 | 0.47+0.01 |
| 7-NI+7-day-tail suspension | 5 (6) | 157.4+6.6 | 50.0+1.7* | 0.31+0.01*^§^ |
| Vehicle+7-day-tail suspension | 4 (4) | 169.7+3.5 | 45.4+2.5* | 0.26+0.01* |

Data concerning transfected muscles were not included.

N = number.

a) N of standardly caged rats corresponds to at least 2 ambulatory rats for each unloading time

b) N of muscles is higher than that of rats, because both muscles were injected with vehicle or AAV

* indicates significant difference vs ambulatory values (ANOVA p<0.001)

§ indicates significant difference vs. sham-infected values (p=0.02) or vehicle-treated ones (p<0.04; ANOVA post-hoc analysis).
