# Supplementary material for: Loss of melusin is a novel, neuronal NO synthase/FoxO3‐independent master switch of unloading‐induced muscle atrophy
Source: J Cachexia Sarcopenia Muscle. 2020 Mar 10;11(3):802–19. doi: 10.1002/jcsm.12546 (PMC7296270; doi:10.1002/jcsm.12546)
Supplement: Supplementary file 7 — Table S2. Primer sets used for qPCR [file JCSM-11-802-s007.doc]

**Supplemental Table 2. Primer sets used for qPCR**

| **Primer** | **Forward (5'-3')** | **Reverse (5'-3')** |
| --- | --- | --- |
| Itgb1bp2 rat | AATTCCAAAGTCAGCAGAGACCTT | GTAGCAGTTTGGGAGGCATCT |
| Gapdh rat | GGAGAAGGCTGGGGCTCA | CTCGTGGTTCACACCCATCAC |
| Atrogin rat | CTACGATGTTGCAGCCAAGA | GGCAGTCGAGAAGTCCAGTC |
| MuRF1 rat | GCCAATTTGGTGCTTTTTGT | AAATTCAGTCCTCTCCCCGT |
| Foxo1 rat | GAGGTGCAATGTGGGAGAAT | TTGAATGAAATGGCAAAGCA |
| Foxo3a rat | TCTCCCGTCAGCCAGTCTAT | AGTCACTGGGGAACTTGTCG |
